# A case report of squamous cell carcinoma mimicking interdigital intertrigo

**DOI:** 10.1093/omcr/omae066

**Published:** 2024-07-09

**Authors:** Ilham Snoussi, Safa Boulifa, Faiçal Abbad, Ouiame El Jouari, Salim Gallouj

**Affiliations:** Department of Dermatology and Venereology, Mohammed VI University Hospital, M3MF+GCG, La Nouvelle Ville Ibn Batouta, Tangier, Morocco; Department of Dermatology and Venereology, Mohammed VI University Hospital, M3MF+GCG, La Nouvelle Ville Ibn Batouta, Tangier, Morocco; Ibn Battouta Laboratory of Anatomic Pathology, Av. Al Qods, Tanger 90060, Morocco; Department of Dermatology and Venereology, Mohammed VI University Hospital, M3MF+GCG, La Nouvelle Ville Ibn Batouta, Tangier, Morocco; Faculty of Medicine and Pharmacy, Abdemalek Essaadi University, M3MF+GCG, La Nouvelle Ville Ibn Batouta, Tangier, Morocco; Department of Dermatology and Venereology, Mohammed VI University Hospital, M3MF+GCG, La Nouvelle Ville Ibn Batouta, Tangier, Morocco; Faculty of Medicine and Pharmacy, Abdemalek Essaadi University, M3MF+GCG, La Nouvelle Ville Ibn Batouta, Tangier, Morocco

**Keywords:** squamous cell carcinoma, interdigital intertrigo, surgery, case report

## Abstract

Squamous cell carcinoma (SCC), also known as epidermoid carcinoma, represents the most common malignant tumor affecting the nails. A 60-year-old tailor with no significant medical history presented with a three-year history of macerated skin between the toes, previously treated with a topical antifungal. Dermatological examination revealed a verrucous, infected ulceration with infiltrated and hyperkeratotic edges, characterized by a whitish, fissured base. This lesion in the fourth interdigital space and extended onto the dorsal surface of the foot without any other associated symptoms. The clinical diagnosis identified it as a neoplastic ulceration. An initial biopsy found keratoacanthoma but showed no malignant features. However, follow-up biopsy at our department revealed moderately differentiated SCC. Surgical resection was successful in treating our patient. Diagnostic errors due to insufficient understanding of the pathology and inadequate biopsy methods contribute to the progression of SCC. Surgery is the main treatment for such malignant tumors.

## Introduction

Squamous cell carcinoma (SCC), also known as epidermoid carcinoma, is the most prevalent malignant tumor impacting skin and nail health [1, 2]. This carcinoma predominantly affects men, accounting for 50% to 75% of cases [1–3]. It primarily occurs in middle-aged individuals, with the highest incidence observed between the ages of 50 and 69 [3]. SCC is commonly found on the fingers and is less frequently seen on the toes. The occurrence of SCC in the interdigital spaces is particularly rare [3]. Interdigital SCC may develop from the progression of prior traumatic or inflammatory lesions, including but not limited to unstable burn scars or chronic ulcers [3, 4]. In this report, we present a case of interdigital SCC arising in a patient with chronic intertrigo.

## Case report

**Figure 1:**
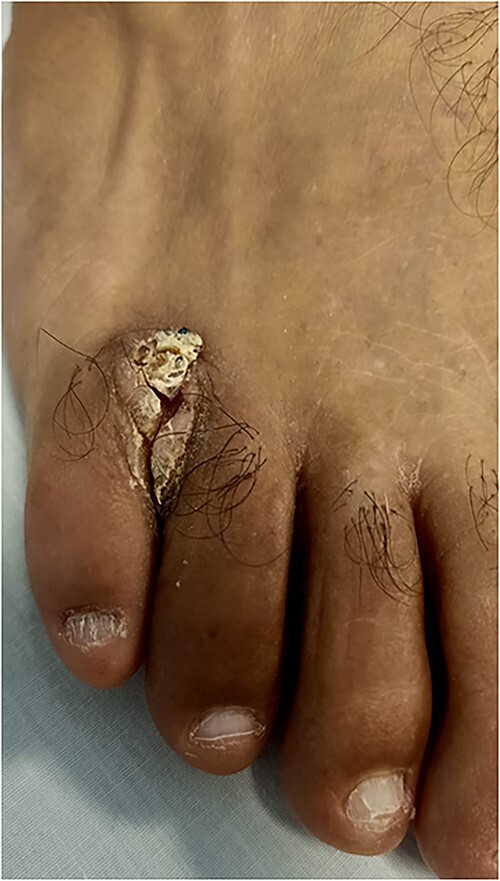
Verrucous ulceration with infiltrated edges in the fourth interdigital space on the right foot, extending onto the dorsum (top) of the foot.

A 60-year-old man, employed as a professional tailor and with no significant medical history, presented with a three-year history of macerated skin between his toes, initially treated with a topical antifungal. He sought medical consultation for a painful, fissured ulceration located at the base of the fourth interdigital space. This ulceration had been developing over the past 18 months and had progressively increased in size. Despite multiple courses of antifungal treatments based on a combination of sertaconazole (2%) topically for 6 weeks and oral terbinafine at a dose of 250 mg (1 tablet per day) for another period of 6 weeks, no improvement was not observed. During the dermatological examination, a verrucous, infected ulceration was observed. It featured infiltrated, hyperkeratotic edges and a whitish, fissured base measuring 3 cm in diameter. The lesion extended onto the dorsal aspect of the foot, without any other accompanying signs (Fig. 1).

**Figure 2:**
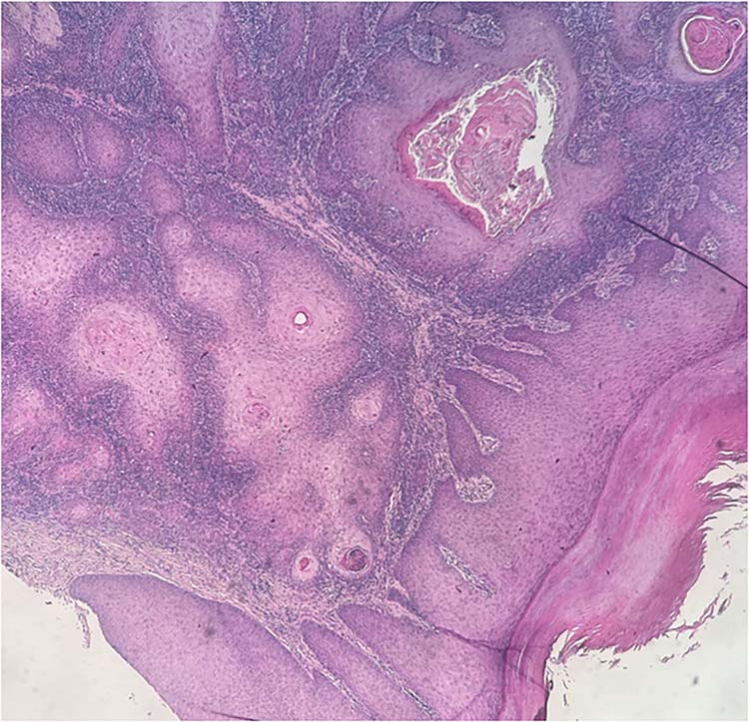
Tumoral proliferation of squamous nature composed of masses and lobules, presence of areas of dyskeratosis (cornoid lamellae)—histological image stained with hematoxylin and eosin (H&E) *4.

**Figure 3:**
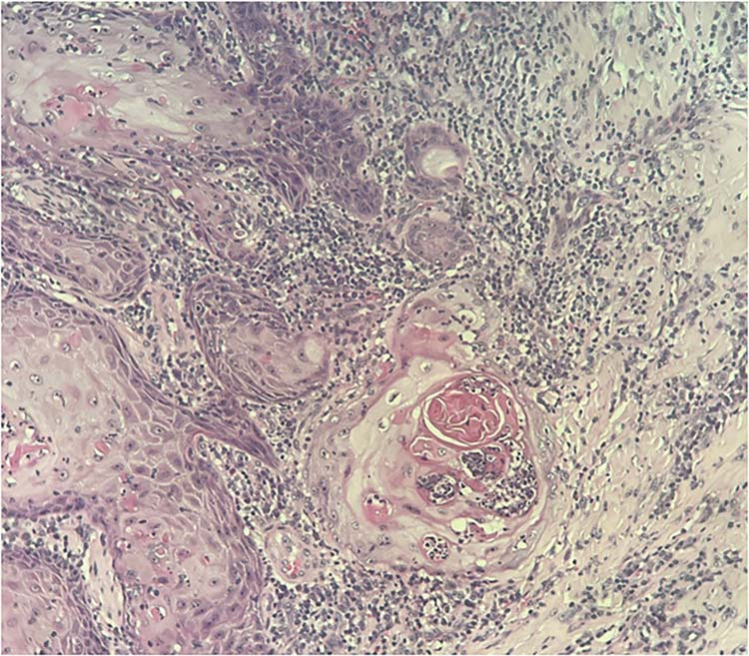
Carcinomatous proliferation of squamous nature (invasive front)—carcinoma cells with nuclei showing severe atypia. Abundant dyskeratotic cytoplasm. Abundant fibro-inflammatory stromal reaction—histological image *40 stained with hematoxylin and eosin (H&E).

**Figure 4:**
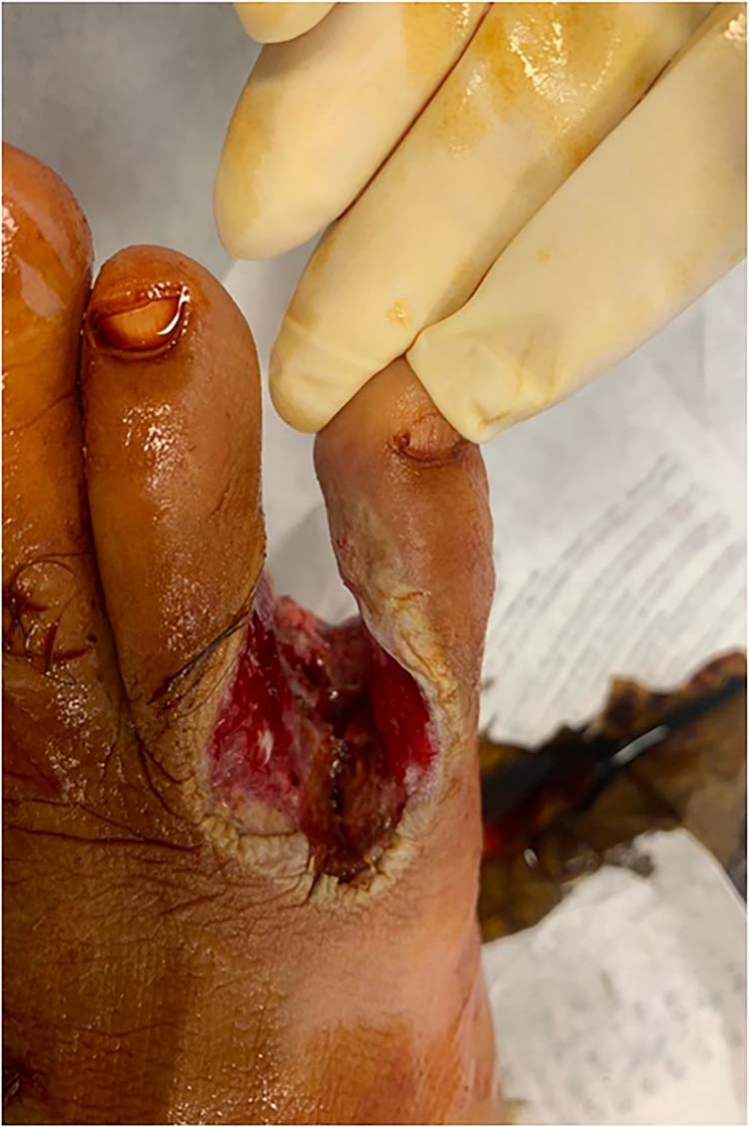
Wide excision of the tumor with a 5 mm safety margin extending to the deep plane.

During dermoscopy, unstructured whitish areas and hyperkeratosis were noted. Lymph node examination, particularly in the popliteal and inguinal regions, revealed no abnormalities. No other significant findings were observed in the comprehensive somatic examination. The clinical diagnosis was a neoplastic ulceration, identified as squamous cell carcinoma (SCC). SCC is a common mimic in dermatology, often mistaken for typical warts. Another differential diagnosis considered was keratoacanthoma. An initial biopsy, conducted externally, indicated keratoacanthoma without malignant features. However, a subsequent biopsy in our department revealed moderately differentiated SCC (Figs 2 and 3). Standard foot radiography showed no osteolytic activity. Chest radiographs (anteroposterior and lateral views) were normal, as was an ultrasound of the lymphatic regions. A wide excision of the lesion, including a 5 mm safety margin and involving the fourth and fifth toes of the right foot, was successfully performed. Histopathological analysis of the excised tissue confirmed moderately differentiated SCC with clear margins (Fig. 4). A follow-up clinical examination for 5 years every 6 months was planned in combination with a lymph node ultrasound every 6 months for 5 years then annually.

## Discussion

This case highlights a rare instance of SCC in a specific and uncommon location [3, 5]. Pathogenetically, chronic interdigital maceration is increasingly recognized as a potential etiological factor in carcinoma development [3]. Such a location can often present a misleading clinical picture, which may lead to a delay in diagnosis [3, 5]. SCC in the interdigital space may appear as persistent macerated intertrigo, resistant to standard treatments [3, 6]. Differential diagnoses may include conditions like corns, inverse psoriasis, or chronic hyperkeratosis, each potentially contributing to SCC onset [7]. Baptista’s seminal work from 1975, which focused on interdigital SCC, documented 22 cases predominantly affecting females [3]. This contrasts with our case, involving a male patient. Typically, these tumors affect the last two interdigital spaces and often originate from chronic fungal intertrigo or corn [1]s, as demonstrated in this case. The interdigital intertrigo in our patient persisted for three years, with chronic maceration being a significant contributory factor to the development of SCC [1, 5], more so than trauma [5].

In the series previously mentioned, this condition was frequently observed among agricultural workers who wore rubber shoes [5]. Similarly, our patient, a tailor, regularly wore ill-fitting shoes and practiced inadequate hygiene, particularly in cleaning the interdigital spaces. SCC in such cases often remains unrecognized in its early stages, being commonly mistaken for simple fungal intertrigo despite multiple medical consultations [4]. To ensure accurate diagnosis, biopsies should be extensive and deep [1], as illustrated in our case where two samples were taken. Due to the initial biopsy yielding inconclusive results, a second biopsy was essential for definitive diagnosis. The preferred treatment method is surgery, involving wide excision [1]. In some cases, this may include the amputation of the two toes adjacent to the affected area [1, 4]. Lymph node dissection is advised only in the presence of palpable satellite lymph nodes [8].

## Conclusion

The diagnostic errors in this case, arising from a limited understanding of the pathology in a clinical setting and inadequate biopsy techniques and interpretation, were associated with the progression of the tumor. The primary modality for treating such cases is based on surgical resection. The rarity of interdigital carcinoma may, in part, be attributed to its underestimation in clinical practice.

## Author contributions

IS wrote the manuscript. SB participated in the management of the patient. FA provided histopathological review. OEJ and SG supervised the case management and writing.

## Conflict of interest

The authors declare that they have no competing interests.

## Funding

This research did not receive any external or internal funding.

## Ethics Approval and Consent to Participate

Ethical review was not required based on local regulations on single cases. The patient provided written consent for using his medical data for research purposes.

## Consent for Publication

The patient provided written consent for using his medical data for research purposes. A copy of the written consent is available for review by the Editor-in-Chief of this journal.

## Data availability

All data and medical information used in this study are available upon reasonable request.
